# Missing Links Between Gene Function and Physiology in Genomics

**DOI:** 10.3389/fphys.2022.815874

**Published:** 2022-02-28

**Authors:** Julio Collado-Vides, Pascale Gaudet, Víctor de Lorenzo

**Affiliations:** ^1^Centro de Ciencias Genómicas, Universidad Nacional Autónoma de México, Cuernavaca, Mexico; ^2^Department of Biomedical Engineering, Boston University, Boston, MA, United States; ^3^Centre for Genomic Regulation, The Barcelona Institute of Science and Technology, Universitat Pompeu Fabra, Barcelona, Spain; ^4^SIB Swiss Institute of Bioinformatics, Swiss-Prot Group, Geneva, Switzerland; ^5^Department of Systems Biology, Centro Nacional de Biotecnología CSIC, Universidad Autónoma de Madrid, Madrid, Spain

**Keywords:** gene ontology, microbial annotations, gene function, challenges and issues, mechanisms and physiology

## Abstract

Knowledge of biological organisms at the molecular level that has been gathered is now organized into databases, often within ontological frameworks. To enable computational comparisons of annotations across different genomes and organisms, controlled vocabularies have been essential, as is the case in the functional annotation classifications used for bacteria, such as MultiFun and the more widely used Gene Ontology. The function of individual gene products as well as the processes in which collections of them participate constitute a wealth of classes that describe the biological role of gene products in a large number of organisms in the three kingdoms of life. In this contribution, we highlight from a qualitative perspective some limitations of these frameworks and discuss challenges that need to be addressed to bridge the gap between annotation as currently captured by ontologies and databases and our understanding of the basic principles in the organization and functioning of organisms; we illustrate these challenges with some examples in bacteria. We hope that raising awareness of these issues will encourage users of Gene Ontology and similar ontologies to be careful about data interpretation and lead to improved data representation.

## Introduction

In the first pages of “The Elements of Chemistry,” Antoine Laurent Lavoisier quoted a philologist (a linguist) of his time, to explain the main goal of his classification, the one of making it possible to simultaneously name a substance and to classify it (Lavoisier, 1790/1965). In biology, naming the properties of genes and classifying them is a task that, with the emergence of genomics, can be gradually connected with multiple higher levels of description up to the level of addressing the understanding of the cell. We know that the genome sequence of an organism does not provide understanding of an organism’s functioning. Moreover, even knowing the function of all genes does not necessarily imply that we can understand the functioning of a whole cell. In this manuscript we will raise questions with examples referring to bacteria; because bacteria are mostly single-cell life forms with smaller genomes (compared to multicellular and multi-organ organisms), they certainly are a good case study for tackling these issues.

Function can be defined as the role played by an element within the system or ensemble it belongs to. Physiology, on the other hand, is that part of biology devoted to explaining the overall function of cells, tissues, and organs, i.e., the outcome of all combined molecular functions, and how these respond to changing conditions. Since the advent of molecular biology and even more recently the easy availability of genome sequencing, our approaches to describe biological systems have become more and more reductionist. It would be highly beneficial to bridge the gap in our knowledge regarding how molecules translate into the function—and malfunction—of an organism, thus connecting gene function with physiology.

### Functional Categorization Versus Understanding

**FIGURE 1:**
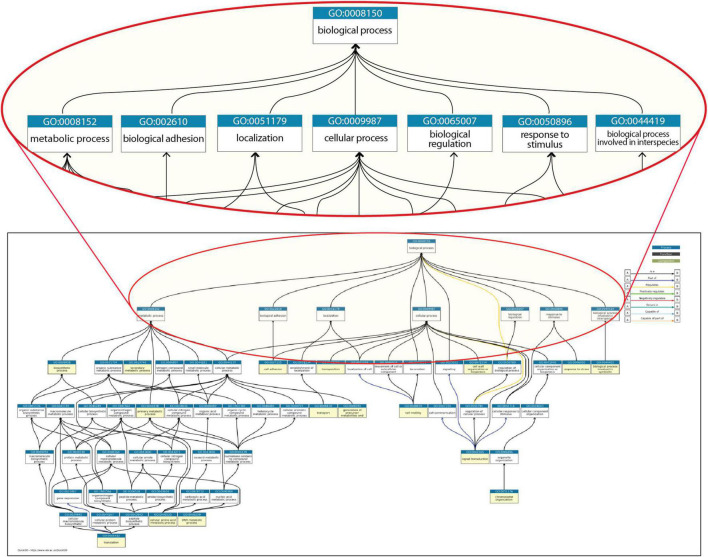
A portion of the structure for “biological processes” within Gene Ontology. All terms shown are required to describe properties of genes from *E. coli* K-12, with an expanded view of the top-level categories. The whole set of categories for *E. coli* K-12 genes can be found at https://biocyc.org/ECOLI/new-image?object=GO%3A0008150.

The major resources, such as Gene Ontology (GO), that capture the annotation of biochemical and functional knowledge of proteins, compounds, reactions, and interactions have made important contributions for genomics (Ashburner et al., 2000; Carbon et al., 2021). GO has enabled a major step forward in genomics by constructing a controlled vocabulary that can be used in the annotation of any molecular process or reaction, in any organism. This is the foundation that supports a large number of tools to perform computation on functional data in the three kingdoms of life. However, as is frequent in life, a strength can pose at the same time a limitation. Figure 1 shows the most commonly annotated top-level terms of the GO categories that are utilized in the description of knowledge of *Escherichia coli* K-12, one of the best-characterized microbes.

At the first level below the root node, “biological process,” there are classes such as cellular processes, biological regulation, response to stimulus, localization, biological adhesion, and metabolic processes. A different classification, MultiFun, devoted to *E. coli*, has 10 major classes: Metabolism, Information Transfer, Regulation, Transport, Cell Processes, Cell Structure, Location, Extra-chromosomal origin, DNA Site, and Cryptic Gene (Riley, 1993). These major categories are the roots of a hierarchy of several subclasses, and individual genes are classified into one or multiple categories in a structure similar to that in Figure 1.

These numerous classes and their subclasses are the result of essentially a bottom-up approach of annotation of individual gene functions across different species within the GO and, in the case of MultiFun, the result of years of work specifically in the annotation of *E. coli* genes by Monica Riley in the earlier days of genome analysis (Riley, 1993; Serres and Riley, 2000). They have the common aim of describing all known functions of gene products. Both GO and MultiFun are examples of classifications used in the annotation of functions of genes as they continue to be uncovered.

While these classifications help in the annotation of gene properties, they are not adequate to fully explain the functioning of an organism. Ideally, the major groupings in a classification system would correspond to the major concepts needed to grasp functional microbiology, or the physiology of a multicellular organism. The prevailing view of a cell is that of an evolving and self-replicating chemical factory run by a program, coded in the DNA, and whose main (if not sole) agenda is to survive and proliferate as much as possible, hence ensuring the transmission of its genome. This involves adapting to a changing and often stressful, competitive environment. This general principle is not conveyed at the high-level description of the functional genomic classifications. Why is there such a large discrepancy between the major classes of functional classification systems and the main concepts required to understand the major principles of microbial physiology?

Function, as defined in GO (Thomas, 2017), is ‘‘a specific objective that the organism is genetically programmed to achieve.’’ Note that this definition encompasses what GO refers to as ‘‘molecular function’’ and ‘‘biological process.’’ The contributions of both annotations for a gene product identify the properties within a larger ‘‘system’’ it belongs to. However, these annotations and classes are too granular for general principles such as fitness, survival, and viability to emerge. These types of concepts, concerning the properties of entire organisms (phenotypes, in the wide meaning of the term), are described in other ontologies such as Ascomycete Phenotype Ontology.^1^ There is no easy way to computationally navigate between these organism-level observations and the complement of reactions and signaling processes that mediate them.

### Granularity of Gene Functions Versus Physiological Processes

The granularity of genes may be too low for understanding where a physiological capability achieved at the molecular level is located in the larger cellular context. The structure of the GO contains three different classifications, defined as: molecular function for the activities of individual genes, biological process for pathways and larger processes also defined as specific objectives that the cell or organism is genetically programmed to achieve, as already mentioned, and cellular component to indicate where the gene products are active^2^.

A less-detailed level of description, to understand processes, could be that of biochemical pathways, or similarly, the level of activities of collections of genes within transcription units, operons, and even at the level of regulons, that is to say, genes subject to regulation by the same transcription factor (TF). An annotation effort at this level is illustrated by the molecular and functional descriptions of regulons or genetic sensory-response units (GENSOR Units) in RegulonDB (Gama-Castro et al., 2016). Take for instance the TrpR (tryptophan regulator) regulon of *E. coli.* In RegulonDB, the collective function of 9 genes is summarized as: ‘‘The presence of tryptophan inhibits the expression of genes needful for transport and synthesis of tryptophan from serine and conversion of erythrose-4P to chorismate, a precursor of tryptophan’’.^3^ This would be called tryptophan homeostasis in GO. This example shows a good direction of succinctly describing genetic capabilities of groups of genes. The Gene Ontology Consortium, with the development of the Causal Activity Model (GO-CAM), now provides a mechanism to express this type of information (Thomas et al., 2019).

We know that only around one-fifth of regulons in *E. coli* K-12 encode genes that collectively belong to a single biological process; several regulons encode genes involved in multiple biological processes, whereas in 16% of the 189 known regulons, the gene products encode reactions that cannot be connected to build pathways (Ledezma-Tejeida et al., 2019).

When grouping genes into complex regulons, defined as those subject to regulation by multiple and exactly the same TFs, the functional homogeneity of their regulated genes increases considerably in terms of belonging to a dominant functional class [See Figure 3 in Ledezma-Tejeida et al. (2019)]. It is true that this has to be taken with a grain of salt, since currently described regulons are not yet comprehensive since new TF-gene interactions may still be discovered for many TFs. We need to wait for the full genome characterization of complex regulons to solidify this interesting observation. Certainly, finding biologically motivated classes of genes that together perform a dominant function would offer a highly desired intermediate granular level of functional description connecting gene functions with cellular capabilities. At present, we can only speculate whether this intermediate level will be first confirmed in *E. coli* K-12, present in other bacteria, and less likely, present also in eukaryotes. It will be important to keep capturing whole-genome TF-gene regulatory interactions. Next-generation functional analysis tools should be able to integrate the various levels of knowledge to allow more powerful querying.

### Dealing With Unknowns

One non-trivial issue is that—logically—we follow a conceptual frame of what a living system is and what is necessary for it to work according to the prevalent concepts and notions of the time. This rules out, therefore, far unknown requirements that may be disclosed in the future when more knowledge is acquired, and it raises the challenge of handling the sure *unknown unknowns* that still remain to be understood and cannot yet be assigned sound gene annotations. In fact, we always work with incomplete knowledge. There is no genome yet with all its genes functionally characterized. GO deals with this by annotating a gene directly to the root term (either “molecular function,” “biological process,” or “cellular component”) when any one aspect has not been characterized. This formally means that the gene *enables some “molecular function,” participates in some “biological process*,” and *is active in some “cellular component.”* Fully annotated organisms have at least this information for every gene.

Another confounding factor is semantic ambiguity, even in the scientific community. Unequivocal functionality/annotation of a gene can be assigned in many cases without ambiguity, especially when the meaning of a word or a concept is clearly defined in a dictionary or an ontology. But the information used to annotate genomes is extracted from research articles using natural language processing, and the meaning of a term used in an article may differ significantly from that of the ontological concept definition, leading to misinterpretation and inconsistencies in annotation.

### Orthology-Based Annotations

A traditional approach to functionally annotate ORFs (open reading frames) on the basis of the function historically assigned a first biological description and then propagated it to related sequences. Without further evidence, this is a reasonable approach to a first “guess” of the function of an uncharacterized gene, but this can lead to confusion, since contradicting that initial misannotation can be difficult, if only because researchers are misled rather than just unguided in their hypothesis.

A revealing example is the function of the arch-famous Catabolite Regulation Protein, or CRP. The *crp* gene was first discovered and exhaustively studied in *E. coli* as a key component of the regulation of the equally arch-famous *lac* promoter in this species. It was later found that CRP also regulates a large number of other metabolic processes, in addition to utilization of lactose as a carbon source, both negatively and positively in response – *inter alia* – to fluctuations in intracellular cAMP levels. Yet, years later it was shown that virtually identical CRP orthologs found in other bacterial species [e.g., *Pseudomonas* (Milanesio et al., 2011)] have nothing whatsoever to do with metabolism but play a role in membrane-related functions. Finally, recent data suggest that in reality CRP in *E. coli* is not only a promoter-specific regulator but also an authentic genome-scaffolding protein (Heyde et al., 2021) which responds not just to cAMP but to other physiological effectors like cytidine (Lauritsen et al., 2021) and thus has an additional global role. This highlights the importance of maintaining ontologies and the databases relying on them up to date to represent as closely as possible the current state of knowledge.

The *crp* gene example is not an isolated case: it illustrates a widespread phenomenon called exaptation, i.e., co-opting of a given biological object (or its gene thereof) for a function very different from what it originally evolved. How to translate such functional ramifications of the same gene into sound criteria for predicting authentic biological roles remains a serious challenge. A conserved mechanism (binding of a TF to a given target DNA in response to a distinct effector) may result in entirely different physiological outcomes. Annotations in genomic databases have traditionally merged molecular mechanisms of individual gene products and their physiological roles-in-context, thereby causing considerable confusion. GO-CAM alleviates this problem, by connecting gene function to activators, substrates, and larger processes.

The Gene Ontology Consortium has been using a phylogenetic-based approach to complement annotations directly derived from experimental data (Gaudet et al., 2011). Conserved orthologs are annotated with functions that are expected to be conserved in the target species, while taking care not to infer functions that can easily diverge following gene duplication. This increases the coverage of annotations, in particular for non-model organism species for which experimental data are more scarce.

### Universal Principles of Microbial Physiology

In his reference book on bacterial physiology, Frederick Neidhardt suggests that the whole set of chemical reactions of bacterial cells can be categorized as: assembly, polymerization, biosynthetic, and fueling reactions (Neidhardt et al., 1990). This, or alternative higher-level descriptions, could help to identify the most conserved elements present in many organisms and facilitate the identification, or even the validation within new genomes, of plausible general principles of cell physiology.

Certainly, in addition to shared evolutionary origin, any substance has both physical and chemical properties that the cell has to deal with, and as a consequence, some aspects of their biochemistry are shared across many organisms. Processing of carbon sources and nitrogen sources happens with associated consequences in acidification or osmotic changes in *E. coli* and in many other bacteria. As a consequence, some aspects of the combinatorial nature of control may also be shared across many bacteria, such as co-occurrence of nitrogen with extrusion of acid, or the use of glucose and extrusion of acid stress; all of these are due to the common chemical ground of molecules and their properties. It is reasonable to assume that some combinations or integration of physiology will be shared across bacteria given some universal properties of such biochemical and chemical processes, even if the precise mechanisms vary from one species to the next. Most likely, shared combinations of physiology will be found in organisms that have commonalities in their environment.

The top nodes of the biological process GO, similar to the top 10 nodes of MultiFun, include categories that are devoid of physiological content, such as: metabolic process, cellular process, biological regulation, signaling, or transport. These ontology-driven concepts contribute to a way of ordering the activities of genes. An interesting link would be to map gene functions to a separate collection of categories, all of which entail physiological content, with a structure reflecting the organization of bacterial physiology. Bacteria are governed by the need to survive, grow, and reproduce. A first exercise would require an annotation effort that could capture the consensus perspective of a group of experts in the field. This conceptual effort would strongly benefit by defining a dialogue with genome-wide and phenotype characterization experiments with tools—similar to gene enrichment—that help both the interpretation of experiments and the improvement of the conceptual model. Briefly, we suggest that a mapping of the current GO categories with a new collection of categories—devoid of ontological requirements—that reflect the priorities of bacterial physiology generated by a group of experts in the field would be a step forward to connect gene categories with principles of microbial physiology.

### Physiology (High-Level) Drives Mechanism (Low-Level) and Not the Other Way Around

It is important to note that the physiological needs of an organism under specific conditions select for given mechanisms available to fulfill the function—and not vice versa. The specific molecular mechanisms to achieve a given physiological function can vary considerably, even among not-so-distant evolutionarily related microorganisms.

A clear-cut boundary between the biology of mechanisms and physiology in our perspective is the distinction between activator and repressors vs. inducible and repressible systems. Terms like activator and repressor in the description of gene regulators are required when describing mechanisms. Furthermore, genes subject to activation can be induced or repressed just as genes subject to repression, in the sense of being negatively regulated, can be induced or repressed. For instance, the classic LacI repressor is a player of an inducible system since, in the absence of glucose, when lactose is present and is incorporated into the cell, allolactose will bind to LacI, provoking its unbinding from the operator sites and inducing transcription of the *lac* operon. In our opinion the terms “inducible” and “repressible systems” as a consequence of the appearance of a signal belong to physiology, whereas the type of regulator characterizes their mechanisms.

Cellular capabilities described as physiological activities are more likely to remain valid definitions as new research continues to expand and new mechanisms are discovered. An example of this challenge can be found in the discussion and update of major concepts of transcriptional regulation in bacteria (Mejía-Almonte et al., 2020). Whether the two levels (physiological, mechanistic) can be automatically distinguished through computational means and retrieved with the same ease that users enjoy with existing annotation platforms remains to be seen (Mejía-Almonte and Collado-Vides, 2019). Probably advanced, dedicated artificial intelligence approaches will have to be developed and adopted for boiling down the plethora of available data to predict useful and significant functionalities.

### Physiology Versus Mechanisms (Molecular Biology)

We envision that ontologies and databases could/would provide data to test general hypotheses, such as “physiology is more conserved than the specific mechanisms to achieve a given function or biological program.” For this to happen biologically major terms that distinguish molecular biology and mechanistic descriptions from physiology need to be more clearly defined and incorporated into formal frameworks.

Biology is a very rich discipline with knowledge that goes from genetics to behavior, from structure to evolution and dynamics. It is noteworthy that this special journal issue addresses ontology and physiology, whereas most microbial genomic databases are still struggling with mechanisms, processes, and genetics (Kanehisa et al., 2017; Karp et al., 2019; Santos-Zavaleta et al., 2019; Ng et al., 2020; Keseler et al., 2021). Genomic databases gather what we can call genotypic properties, where activities of biomolecules are described but, so far, in the case of microbial organisms, without the precise description of the growing conditions under which a subset of genotypic properties are being actively used.

Gene ontology is a highly used resource to compare pairs of experimental and control whole-genome transcriptional profiles to identify the enriched functions within the genes that show changed expression. The expanding genomic high-throughput technologies are accelerating the identification of the elements of transcriptional regulatory networks (transcription start sites, transcription factor binding sites, terminators, transcription units, and expression profiles) at the genomic level. How can we best combine these resources to characterize the dynamics of inducing and repressing complete biological processes and link them to their mechanisms? Combining genomes, functional gene annotations, and the deciphering of regulatory networks should improve our capability of mapping specific mechanisms with cellular collections of physiological capabilities.

### Phenotypic High-Throughput Annotations?

The link genotype-phenotype has been most often assigned on the basis of heterogeneous experiments with data expressed in formats that typically lack semantic let alone quantitative standards amenable to computation. One ongoing trend to tackle this issue is massive testing of mutant collections grown under a large number of standardized culture conditions (nutrients, stresses, etc.). The so-called phenotypic microarrays (Bochner et al., 2001), along with available high-throughput analyses of transcriptomics, proteomics, and metabolomics of the corresponding strains are increasingly establishing the field of phenomics (Acin-Albiac et al., 2020). Phenomics attempts to assign functionalities to given genes on the basis of a data landscape much wider than the typical reductionist approach and linear logic of traditional genetics. Extensive analyses of this type are already available for *E. coli* (Nichols et al., 2011; Otsuka et al., 2015) and are quickly expanding to other species of interest. It comes as no surprise that phenomics has become a fertile target of machine learning approaches (Lürig et al., 2021), applicable to both wet datasets and text sources (Mao et al., 2016) in published literature. These new perspectives (which may also involve considerable investments in technological platforms) have the potential to change the way automated annotations and GOs will be established (Gkoutos et al., 2012) in the not-so-distant future.

## Conclusion

As mentioned in the introduction, we imagine a continuum from individual functions of gene products into a multilayer architecture of categories that can eventually contribute to the understanding of the whole cell’s physiology. It should be clear that, although many examples have been related to GO, we did not aim to focus on limitations of precise ontologies or conceptual models, instead, we offer food for thought to connect step- by-step individual gene annotations with major conceptual frameworks of functioning of cells and bacteria.

We are well aware of the limited nature of this work. For instance, we have relied on one definition of function: the one we used corresponds to the so-called “causal role function,” whereas the “selected effect function” definition points to its evolutionary origin (Thomas, 2017).

The amount of work behind the current biological processes and functions has been immensely productive for classifying genes according to specific and easy-to-comprehend categories. But as biology moves toward a higher-level understanding of living systems, these classifications turn out to be insufficient to grasp what is going on—and new criteria for functional annotations become needed. In fact, we still know very little of how genes and functions are involved in such system-level functioning of the software and hardware of cells (Danchin, 2009) or even to figure out what such “major principles” are. More precise answers to such questions may come from computational resources rich in annotations, similar to ontologies and databases, that are flexible enough to evaluate different models of what a cell is and to test in quantitative terms hypotheses such as “physiology is more conserved than mechanisms,” and other similar generalities.

It is known that microbial processes are quite poorly represented in GO compared to those for eukaryotic, multicellular organisms; this is no surprise given the origin of the GO Consortium, precisely within eukaryotic organisms (Tyler, 2009). Some potential avenues to fill the missing links of physiological categories in bacterial systems include higher-level descriptions, for instance as different modules (i.e., carbon source module; nitrogen source module; acid stress module, etc.) with their defined interactions, where the major objectives of the genetic programs of cells become explicit. Modeling their dynamic behavior as a consequence of changes in growth conditions with the regulatory machinery pointing to specific mechanisms of gene regulation would advance the mapping of the genotype to its physiological capabilities and their flexibility in bacteria.

Defining or even formalizing different types of relations and concepts clearly distinguishing mechanistic, physiological, and evolutionary territories should help in clarifying the complex structure of biological knowledge.

## Data Availability Statement

The original contributions presented in the study are included in the article/supplementary material, further inquiries can be directed to the corresponding author.

## Author Contributions

JC-V, PG, and VL participated in the discussions and in the writing of the ideas presented in the manuscript. All authors contributed to the article and approved the submitted version.

## Conflict of Interest

The authors declare that the research was conducted in the absence of any commercial or financial relationships that could be construed as a potential conflict of interest.

## Publisher’s Note

All claims expressed in this article are solely those of the authors and do not necessarily represent those of their affiliated organizations, or those of the publisher, the editors and the reviewers. Any product that may be evaluated in this article, or claim that may be made by its manufacturer, is not guaranteed or endorsed by the publisher.

## Footnotes

1
https://www.yeastgenome.org/ontology/phenotype/ypo#network

2
http://geneontology.org/docs/ontology-documentation/

3
http://regulondb.ccg.unam.mx/gensorunit?term=GU0000127697&organism=ECK12&format=jsp&type=gensorunit

## References

[B1] Acin-Albiac M. Filannino P. Gobbetti M. Di Cagno R. (2020). Microbial high throughput phenomics: the potential of an irreplaceable omics. *Comput. Struct. Biotechnol. J.* 18 2290–2299. 10.1016/j.csbj.2020.08.010 32994888 PMC7490730

[B2] Ashburner M. Ball C. A. Blake J. A. Botstein D. Butler H. Cherry J. M. (2000). Gene ontology: tool for the unification of biology. The Gene Ontology Consortium. *Nat. Genet.* 25 25–29. 10.1038/75556 10802651 PMC3037419

[B3] Bochner B. R. Gadzinski P. Panomitros E. (2001). Phenotype microarrays for high-throughput phenotypic testing and assay of gene function. *Genome Res.* 11 1246–1255. 10.1101/gr.186501 11435407 PMC311101

[B4] Carbon S. Douglass E. Good B. M. Unni D. R. Harris N. L. Mungall C. J. (2021). The Gene Ontology resource: enriching a GOld mine. *Nucleic Acids Res.* 49 D325–D334. 10.1093/nar/gkaa1113 33290552 PMC7779012

[B5] Danchin A. (2009). Bacteria as computers making computers. *FEMS Microbiol. Rev.* 33 3–26. 10.1111/j.1574-6976.2008.00137.x 19016882 PMC2704931

[B6] Gama-Castro S. Salgado H. Santos-Zavaleta A. Ledezma-Tejeida D. Muñiz-Rascado L. García-Sotelo J. S. (2016). RegulonDB version 9.0: high-level integration of gene regulation, coexpression, motif clustering and beyond. *Nucleic Acids Res.* 44 D133–D143. 10.1093/nar/gkv1156 26527724 PMC4702833

[B7] Gaudet P. Livstone M. S. Lewis S. E. Thomas P. D. (2011). Phylogenetic-based propagation of functional annotations within the Gene Ontology consortium. *Brief. Bioinform.* 12 449–462. 10.1093/bib/bbr042 21873635 PMC3178059

[B8] Gkoutos G. V. Schofield P. N. Hoehndorf R. (2012). Computational tools for comparative phenomics: the role and promise of ontologies. *Mamm. Genome* 23 669–679. 10.1007/s00335-012-9404-4 22814867 PMC3488439

[B9] Heyde S. A. H. Frendorf P. O. Lauritsen I. Nørholm M. H. H. (2021). Restoring global gene regulation through experimental evolution uncovers a NAP (Nucleoid-Associated Protein)-like behavior of Crp/Cap. *mBio* 12:e0202821. 10.1128/mBio.02028-21 34700380 PMC8546631

[B10] Kanehisa M. Furumichi M. Tanabe M. Sato Y. Morishima K. (2017). KEGG: new perspectives on genomes, pathways, diseases and drugs. *Nucleic Acids Res.* 45 D353–D361. 10.1093/nar/gkw1092 27899662 PMC5210567

[B11] Karp P. D. Billington R. Caspi R. Fulcher C. A. Latendresse M. Kothari A. (2019). The BioCyc collection of microbial genomes and metabolic pathways. *Brief. Bioinform.* 20 1085–1093. 10.1093/bib/bbx085 29447345 PMC6781571

[B12] Keseler I. M. Gama-Castro S. Mackie A. Billington R. Bonavides-Martínez C. Caspi R. (2021). The EcoCyc database in 2021. *Front. Microbiol.* 12:711077. 10.3389/fmicb.2021.711077 34394059 PMC8357350

[B13] Lauritsen I. Frendorf P. O. Capucci S. Heyde S. A. H. Blomquist S. D. Wendel S. (2021). Temporal evolution of master regulator Crp identifies pyrimidines as catabolite modulator factors. *Nat. Commun.* 12:5880. 10.1038/s41467-021-26098-x 34620864 PMC8497467

[B14] Lavoisier, A. L. (1790/1965). *Elements of Chemistry.* Mineola, NY: Dover Publications.

[B15] Ledezma-Tejeida D. Altamirano-Pacheco L. Fajardo V. Collado-Vides J. (2019). Limits to a classic paradigm: most transcription factors in *E. coli* regulate genes involved in multiple biological processes. *Nucleic Acids Res.* 47 6656–6667. 10.1093/nar/gkz525 31194874 PMC6649764

[B16] Lürig M. D. Donoughe S. Svensson E. I. Porto A. Tsuboi M. (2021). Computer vision, machine learning, and the promise of phenomics in ecology and evolutionary biology. *Front. Ecol. Evol.* 9:642774. 10.3389/fevo.2021.642774

[B17] Mao J. Moore L. R. Blank C. E. Wu E. H. Ackerman M. Ranade S. (2016). Microbial phenomics information extractor (MicroPIE): a natural language processing tool for the automated acquisition of prokaryotic phenotypic characters from text sources. *BMC Bioinformatics* 17:528. 10.1186/s12859-016-1396-8 27955641 PMC5153691

[B18] Mejía-Almonte C. Busby S. J. W. Wade J. T. van Helden J. Arkin A. P. Stormo G. D. (2020). Redefining fundamental concepts of transcription initiation in bacteria. *Nat. Rev. Genet.* 21 699–714. 10.1038/s41576-020-0254-8 32665585 PMC7990032

[B19] Mejía-Almonte C. Collado-Vides J. (2019). “Towards the prokaryotic regulation ontology: an ontological model to infer gene regulation physiology from mechanisms in bacteria,” in *Proceedings of the 11th International Joint Conference on Knowledge Discovery, Knowledge Engineering and Knowledge Management (IC3K 2019)* (Setúbal: SciTePress), 495–499.

[B20] Milanesio P. Arce-Rodríguez A. Muñoz A. Calles B. de Lorenzo V. (2011). Regulatory exaptation of the catabolite repression protein (Crp)-cAMP system in *Pseudomonas putida*. *Environ. Microbiol.* 13 324–339. 10.1111/j.1462-2920.2010.02331.x 21281420

[B21] Neidhardt F. Ingraham J. L. Schaechter M. (1990). *Physiology of the Bacterial Cell. A Molecular Approcah.* Sunderland, MA: Sinauer Associates Inc.

[B22] Ng P. C. Wong E. D. MacPherson K. A. Aleksander S. Argasinska J. Dunn B. (2020). Transcriptome visualization and data availability at the *Saccharomyces* genome database. *Nucleic Acids Res.* 48 D743–D748. 10.1093/nar/gkz892 31612944 PMC7061941

[B23] Nichols R. J. Sen S. Choo Y. J. Beltrao P. Zietek M. Chaba R. (2011). Phenotypic landscape of a bacterial cell. *Cell* 144 143–156. 10.1016/j.cell.2010.11.052 21185072 PMC3060659

[B24] Otsuka Y. Muto A. Takeuchi R. Okada C. Ishikawa M. Nakamura K. (2015). GenoBase: comprehensive resource database of *Escherichia coli* K-12. *Nucleic Acids Res.* 43 D606–D617. 10.1093/nar/gku1164 25399415 PMC4383962

[B25] Riley M. (1993). Functions of the gene products of *Escherichia coli*. *Microbiol. Rev.* 57 862–952. 10.1128/mmbr.57.4.862-952.1993 7508076 PMC372942

[B26] Santos-Zavaleta A. Salgado H. Gama-Castro S. Sánchez-Pérez M. Gómez-Romero L. Ledezma-Tejeida D. (2019). RegulonDB v 10.5: tackling challenges to unify classic and high throughput knowledge of gene regulation in *E. coli* K-12. *Nucleic Acids Res.* 47 D212–D220. 10.1093/nar/gky1077 30395280 PMC6324031

[B27] Serres M. H. Riley M. (2000). MultiFun, a multifunctional classification scheme for *Escherichia coli* K-12 gene products. *Microb. Comp. Genomics* 5 205–222. 10.1089/omi.1.2000.5.205 11471834

[B28] Thomas P. D. (2017). The Gene Ontology and the meaning of biological function. *Methods Mol. Biol.* 1446 15–24. 10.1007/978-1-4939-3743-1_2 27812932 PMC6438694

[B29] Thomas P. D. Hill D. P. Mi H. Osumi-Sutherland D. Van Auken K. Carbon S. (2019). Gene Ontology causal activity modeling (GO-CAM) moves beyond GO annotations to structured descriptions of biological functions and systems. *Nat. Genet.* 51 1429–1433. 10.1038/s41588-019-0500-1 31548717 PMC7012280

[B30] Tyler B. M. (2009). Viewing the microbial world through the lens of the Gene Ontology. *Trends Microbiol.* 17 259–261. 10.1016/j.tim.2009.05.002 19577929

